# Supplementary material for: Solvent control of water O−H bonds for highly reversible zinc ion batteries
Source: Nat Commun. 2023 May 11;14:2720. doi: 10.1038/s41467-023-38384-x (PMC10175258; doi:10.1038/s41467-023-38384-x)
Supplement: Supplementary file 1 — Supplementary Information [file 41467_2023_38384_MOESM1_ESM.pdf]

Supplementary Materials for

Solvent control of water O−H bonds for highly reversible zinc ion batteries

Yanyan Wang[1] †, Zhijie Wang[1] †, Wei Kong Pang[2], Wilford Lie[3], Jodie A. Yuwono[1,4], Gemeng Liang[1], Sailin Liu[1], Anita M. D'Angelo[5], Jiaojiao Deng[6], Yameng Fan[2], Kenneth Davey[1], Baohua Li[6] *, and Zaiping Guo[1] *

*Corresponding authors. Email: zaiping.guo@adelaide.edu.au; libh@mail.sz.tsinghua.edu.cn

This PDF file includes:

Supplementary Figs. S1 to S41.
Supplementary Table. S1 to S4.

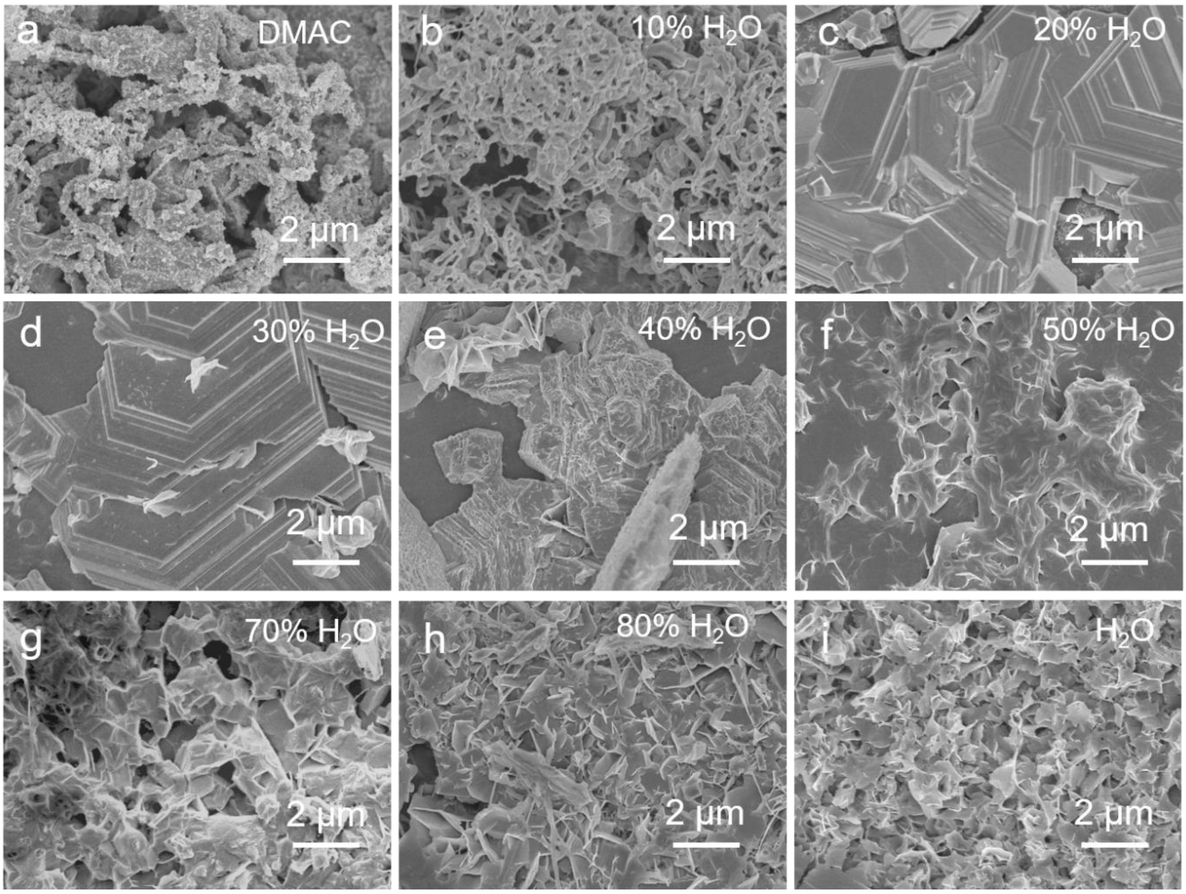

Supplementary Fig. S1. Morphology evolution of Zn deposited in 1 M $Zn(OTf)_2$ solutions. The solvent is a DMAC/H_2O mixture with various volume percentages of H_2O.

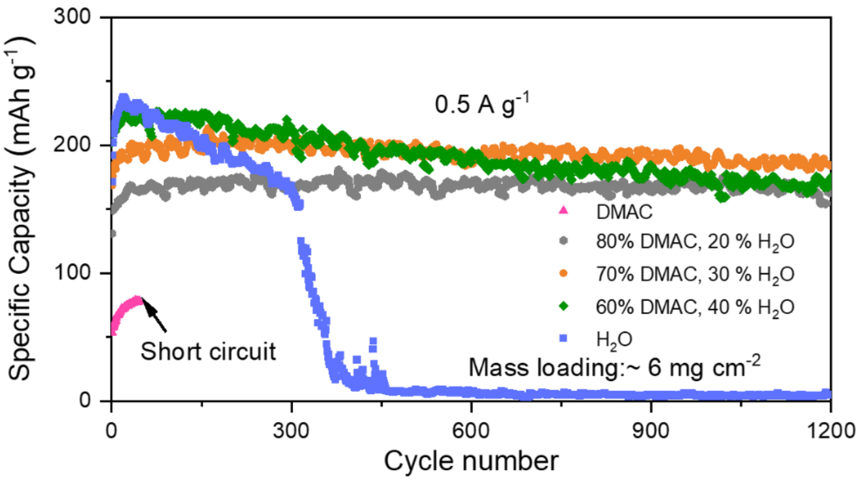

Supplementary Fig. S2. The cyclic performance of Zn||NVO cells in 1 M $Zn(OTf)_2$ solutions. The solvent is DMAC/H_2O mixture with various volume percentages of H_2O.

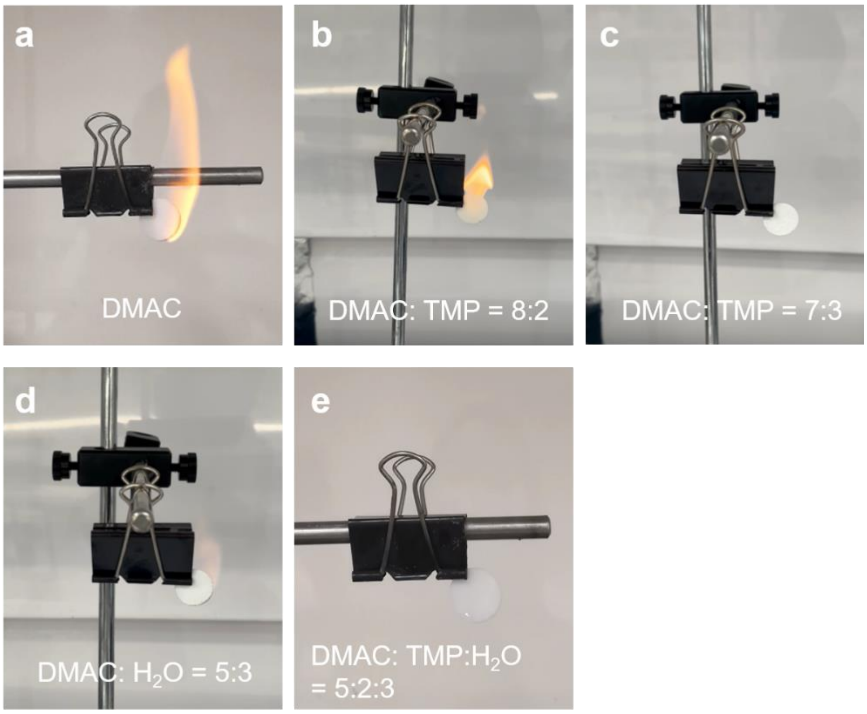

Supplementary Fig. S3. Digital images of ignition test for 1 M $Zn(OTf)_2$ solutions. The solvent is (a) DMAC, (b) DMAC/TMP (8:2 by volume); (c) DMAC/TMP (7:3 by volume); (d) DMAC/H_2O (5:3 by volume); (e) DMAC/TMP /H_2O (5:2:3 by volume).

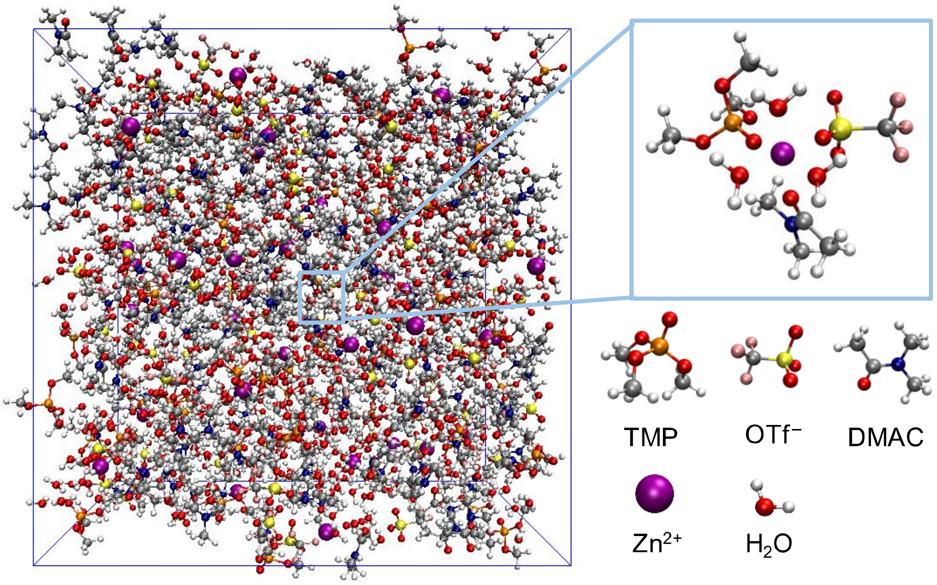

Supplementary Fig. S4. Snapshots of MD simulation boxes for new hybrid electrolyte (HE).

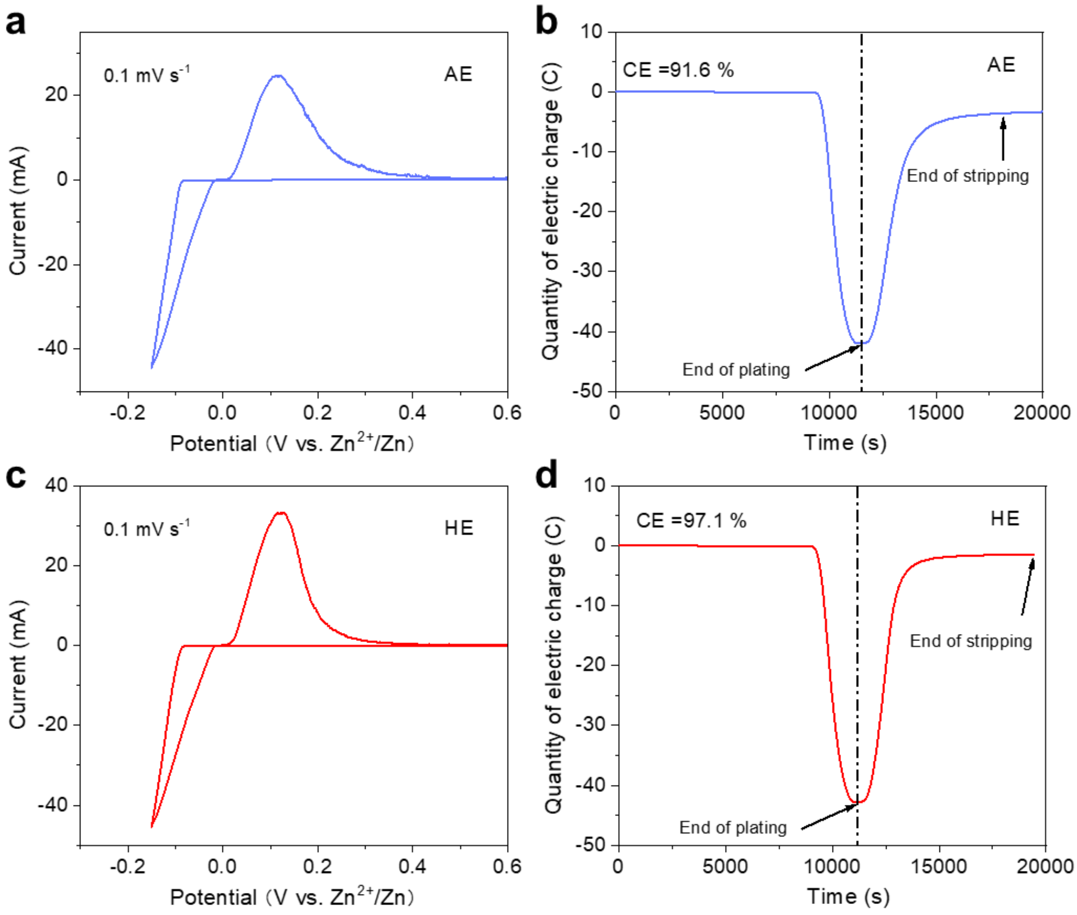

Fig. S5. 3-electrode CV curves and the corresponding CE of Zn plating/stripping in (a-b) AE and (c-d) HE. The working electrode is Cu foil, couter and reference elecyrode is Zn foil.

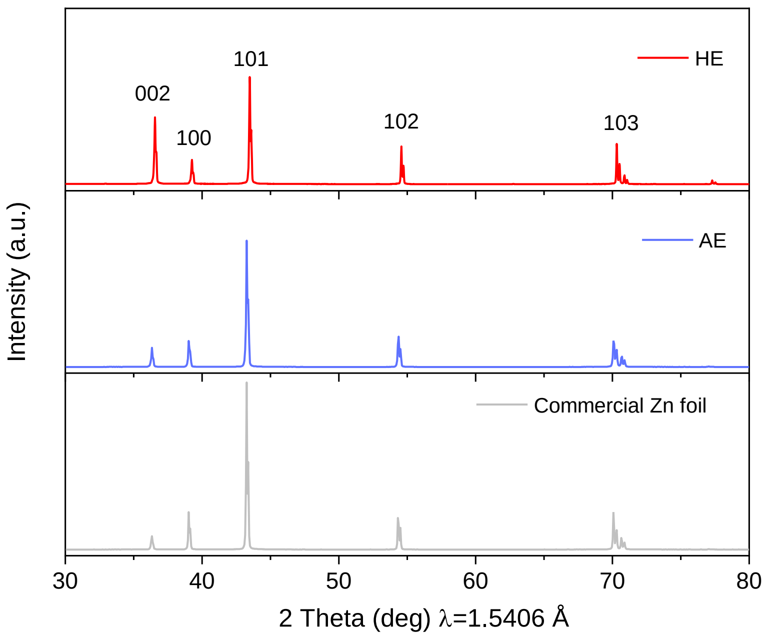

Supplementary Fig. S6. The XRD patterns for commercial Zn-foil, Zn deposited in AE and in HE.

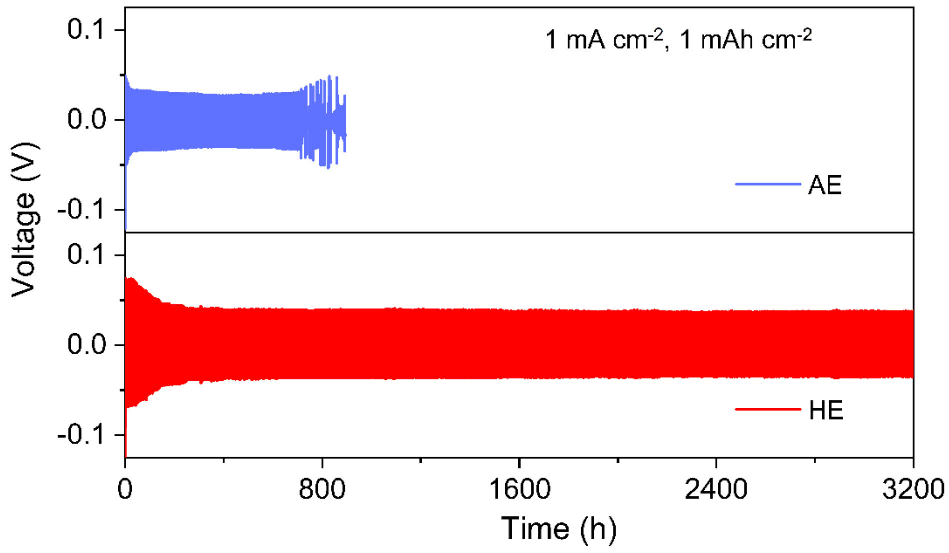

Supplementary Fig. S7. Voltage profile for Zn||Zn symmetric cell working in AE and HE, respectively. Applied current density is 1 mA cm^{-2} and plating capacity 1 mAh cm^{-2}.

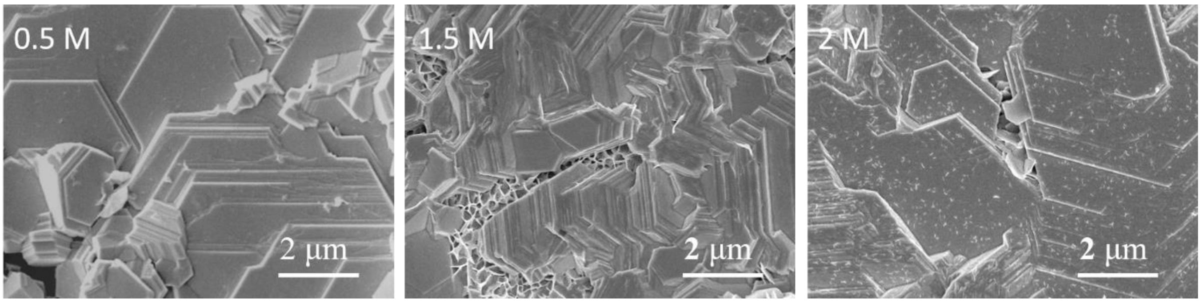

Supplementary Fig. S8. Surface morphology for deposited Zn in hybrid electrolyte with differing $Zn(OTf)_2$ concentration. Solvents are DMAC/TMP/H_2O in volume ratio of 5:2:3.

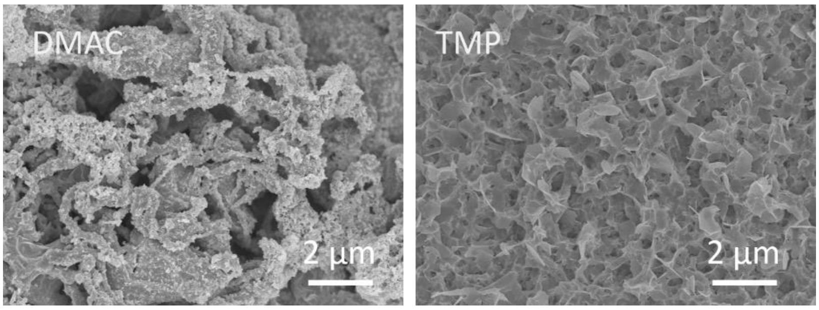

Supplementary Fig. S9. Surface morphology for Zn deposited in 1 M $Zn(OTf)_2$ with DMAC and TMP as single solvent.

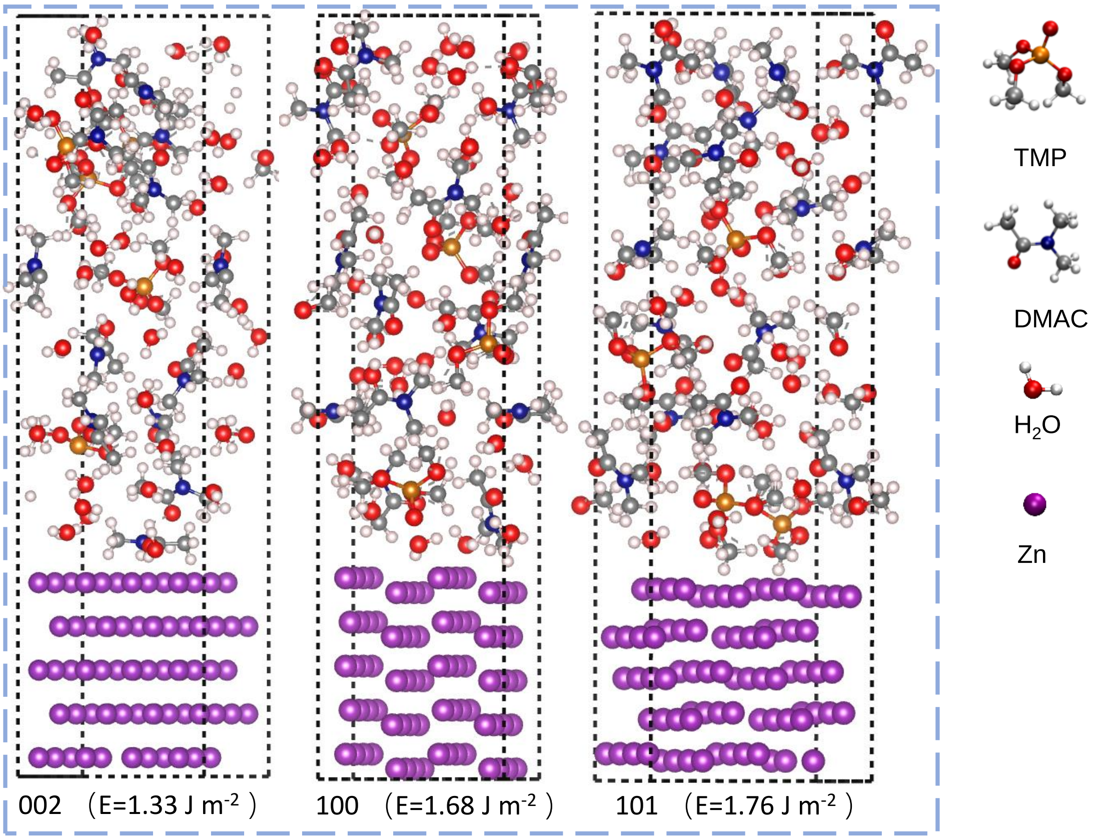

Supplementary Fig. S10. Surface energy for (002), (100) and (101) plane of metallic Zn when exposed in mix of DMAC/TMP/H_2O (5:2:3 by volume).

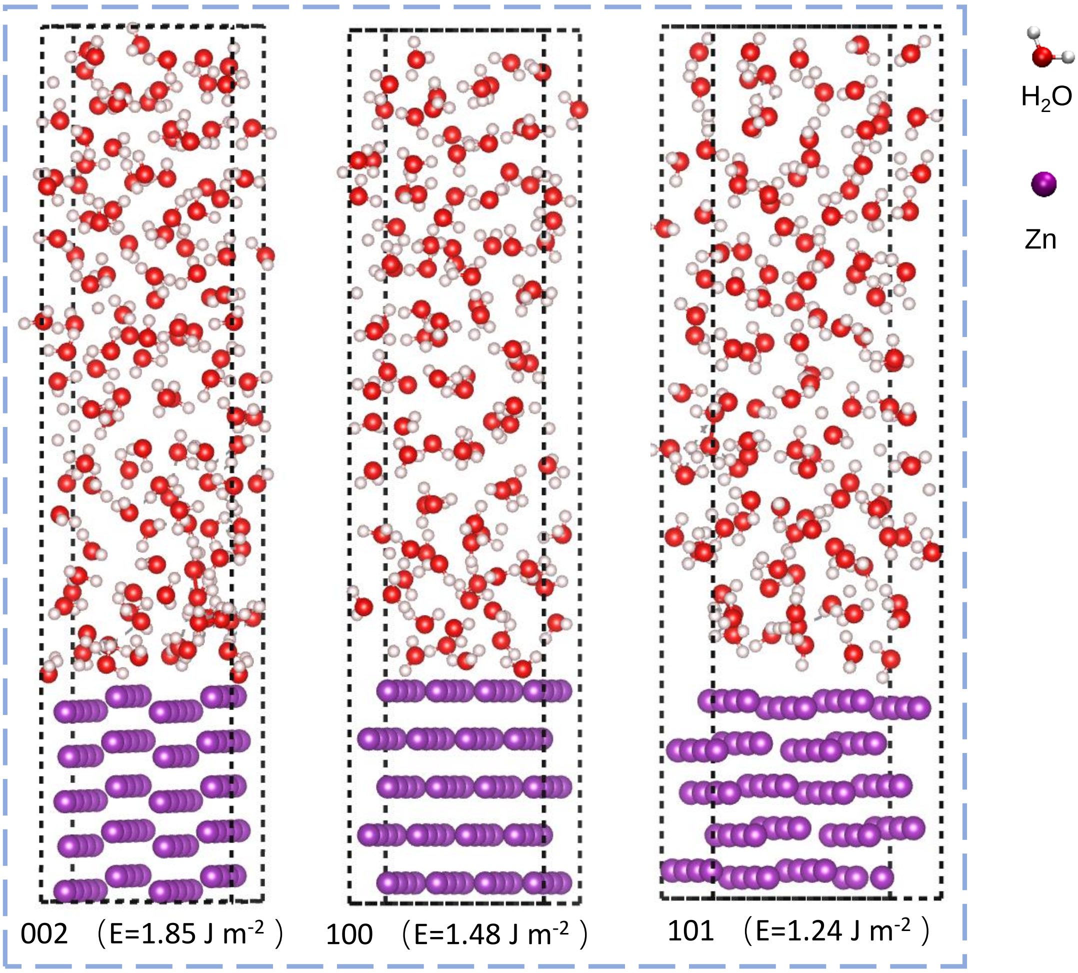

Supplementary Fig. S11. Surface energy for (002), (100) and (101) plane of metallic Zn when exposed in pure H_2O.

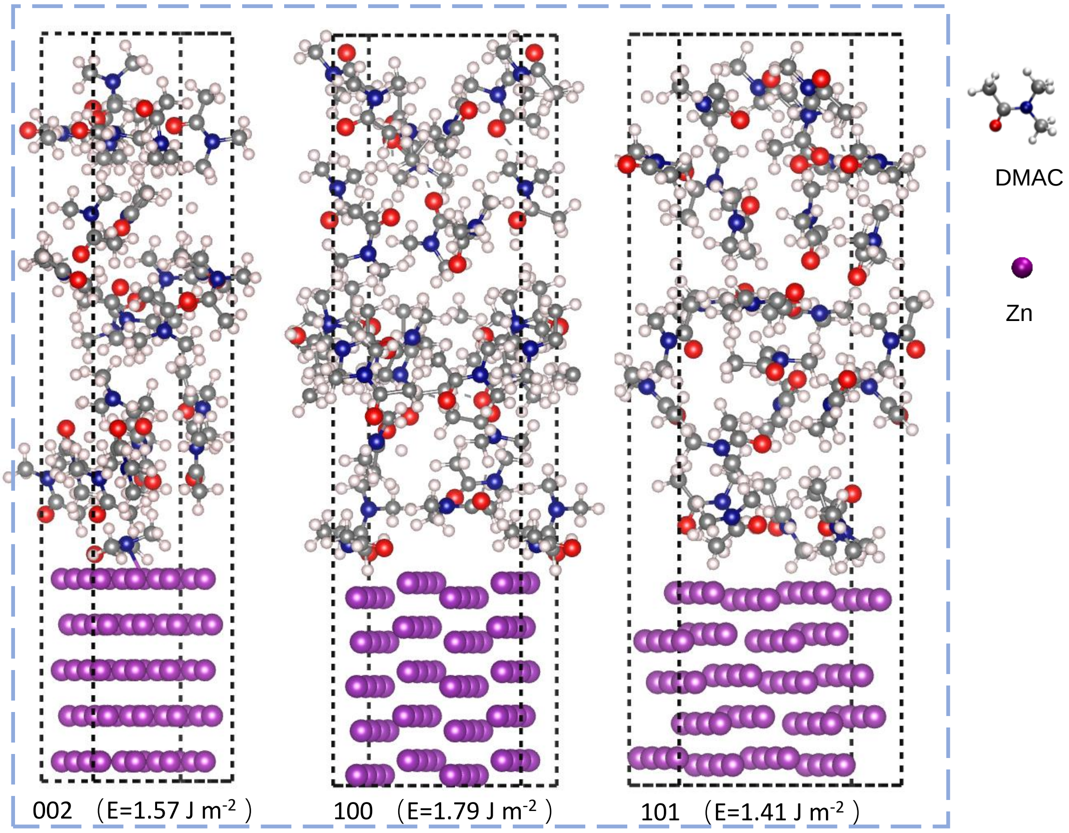

Supplementary Fig. S12. Surface energy for (002), (100) and (101) plane of metallic Zn when exposed in DMAC.

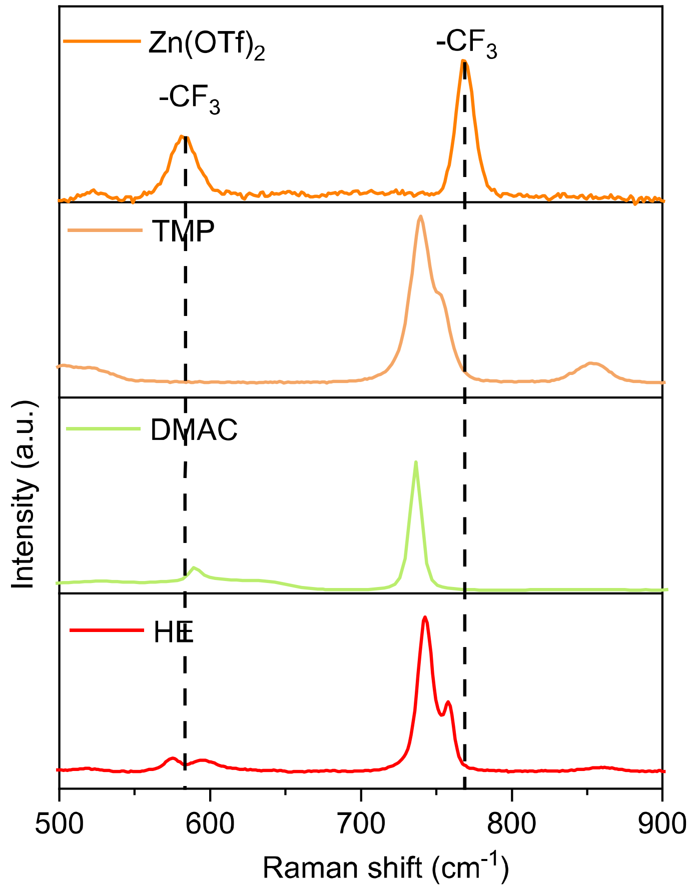

Supplementary Fig. S13. Raman spectra for $Zn(OTf)_2$, TMP, DMAC and HE.

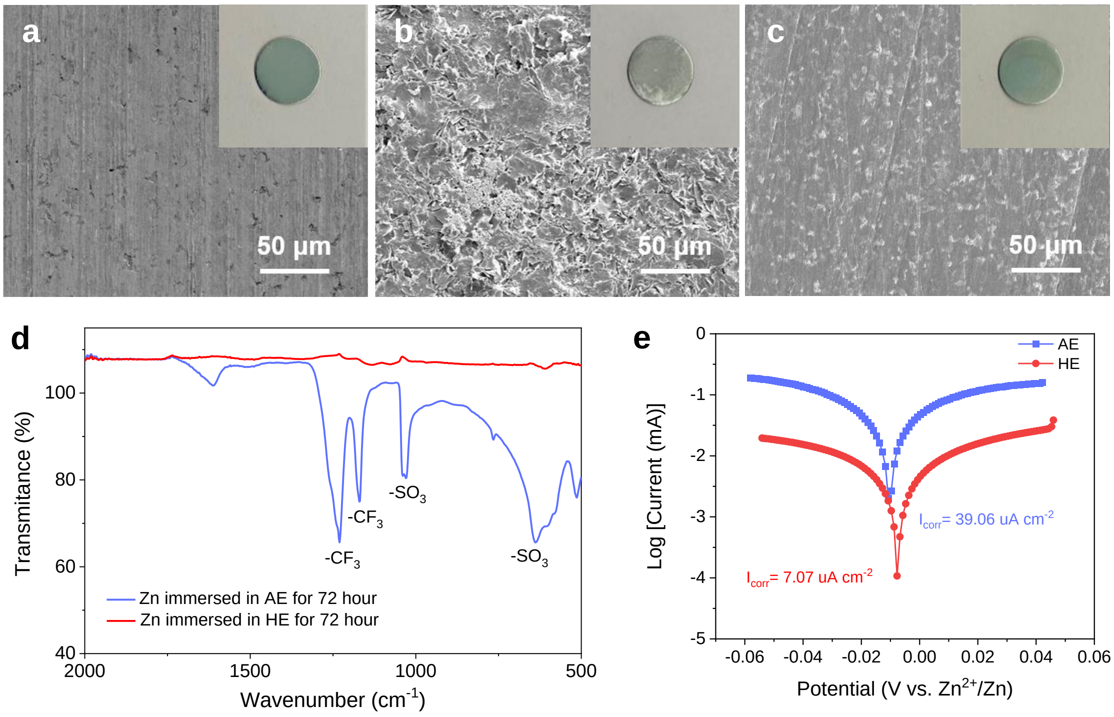

Supplementary Fig. S14. SEM images of (a) fresh Zn-foil and these immersed in AE (b) and (c) HE for 72 h. (d) FTIR spectra for Zn-foil immersed in AE and HE. (e) Tafel curves and corresponding corrosion current density for Zn anode in AE and HE. To avoid interference of Zn deposition, Zn^{2+} ion in AE and HE is replaced with Li^{+} ion.

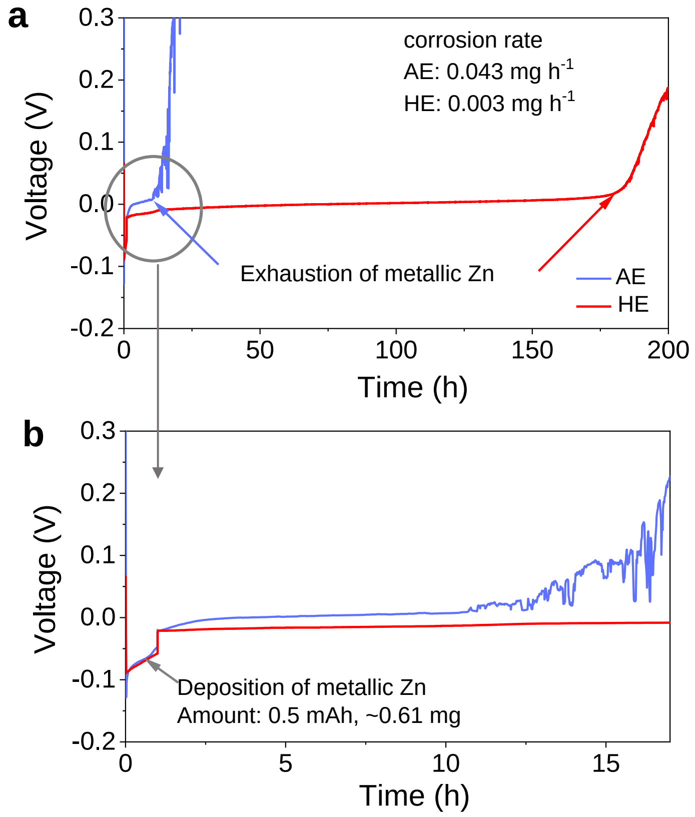

Supplementary Fig. S15. Voltage-time curves for Zn@Ti electrodes immersed in AE and HE, respectively. An amount of metallic Zn, 0.5 mAh, was deposited on Ti-foil to form Zn@Ti electrode.

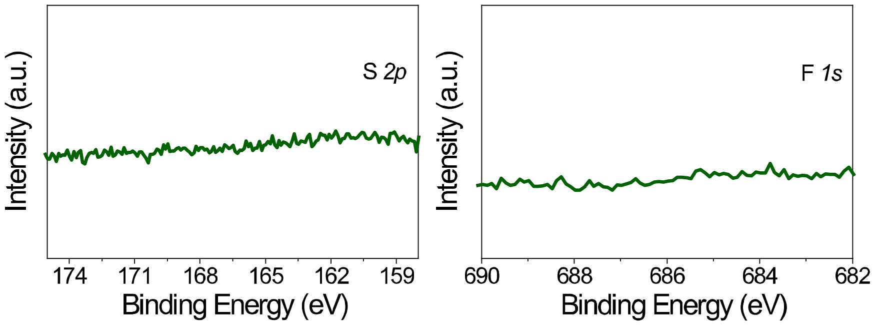

Supplementary Fig. S16. High-resolution XPS spectra for Zn-foil following cycling 100 times with 1 M $Zn(OTf)_2$ in DMAC as electrolyte.

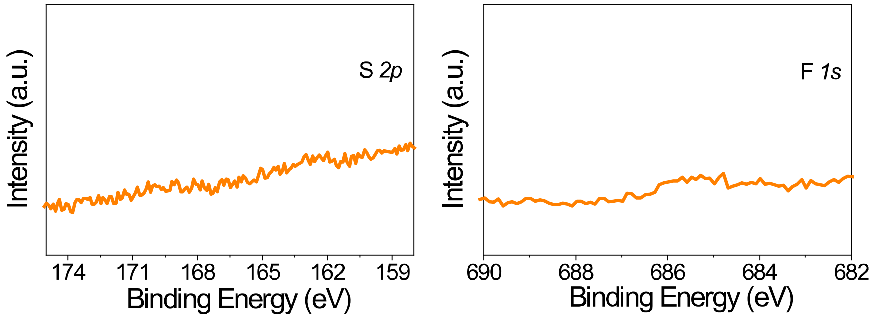

Supplementary Fig. S17. High-resolution XPS spectra for Zn-foil following cycling 100 times with 1 M $Zn(OTf)_2$ in TMP as electrolyte.

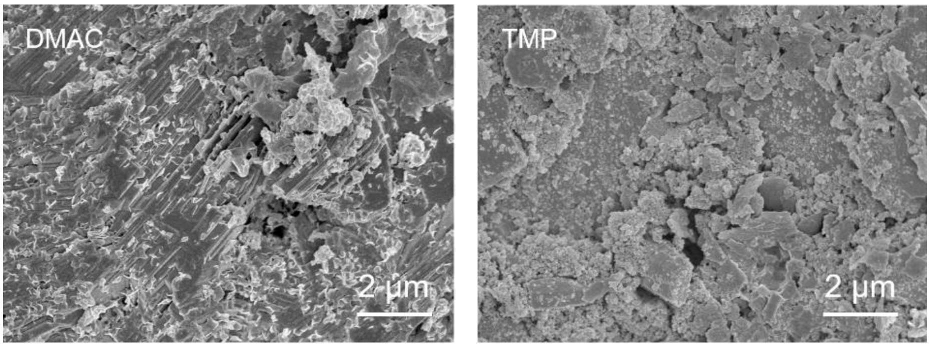

Supplementary Fig. S18. Surface morphology for Zn following 100 cycles in 1 M $Zn(OTf)_2$ with DMAC and TMP as single solvent.

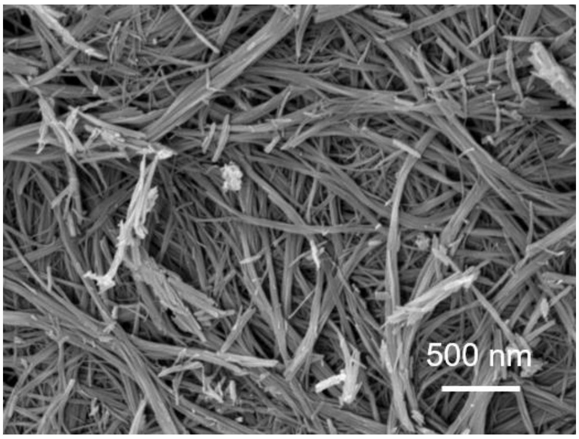

Supplementary Fig. S19. SEM image of NVO powders.

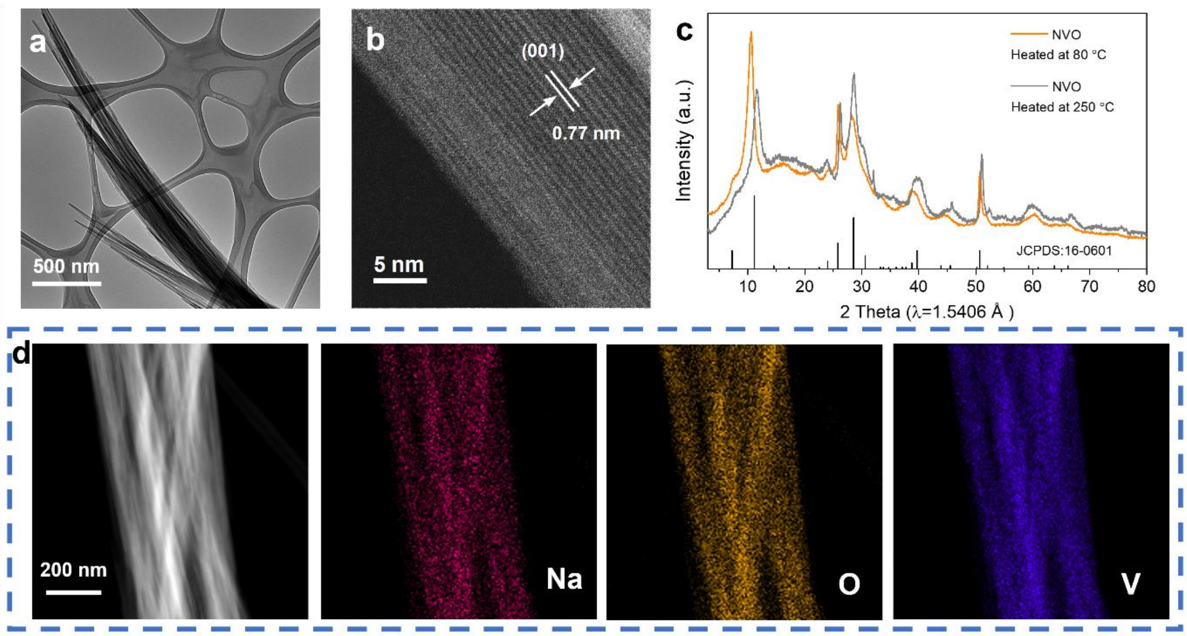

Supplementary Fig. S20. (a) The TEM image of NVO nanobelt; (b) The high-resolution TEM image; (c) XRD patterns of 80 ℃ heated and 250 ℃ heated NVO; (d) TEM elemental mapping images of NVO nanobelt.

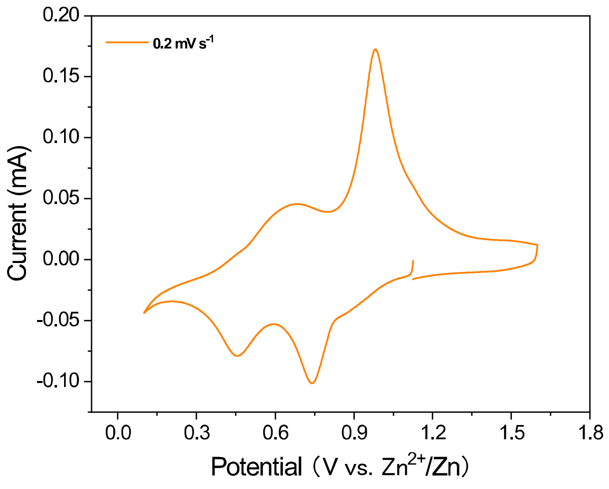

Supplementary Fig. S21. 3-electrode CV curve of NVO electrode scanned at a voltage between 0.1 V to 1.6 V vs. Zn^{2+}/Zn.

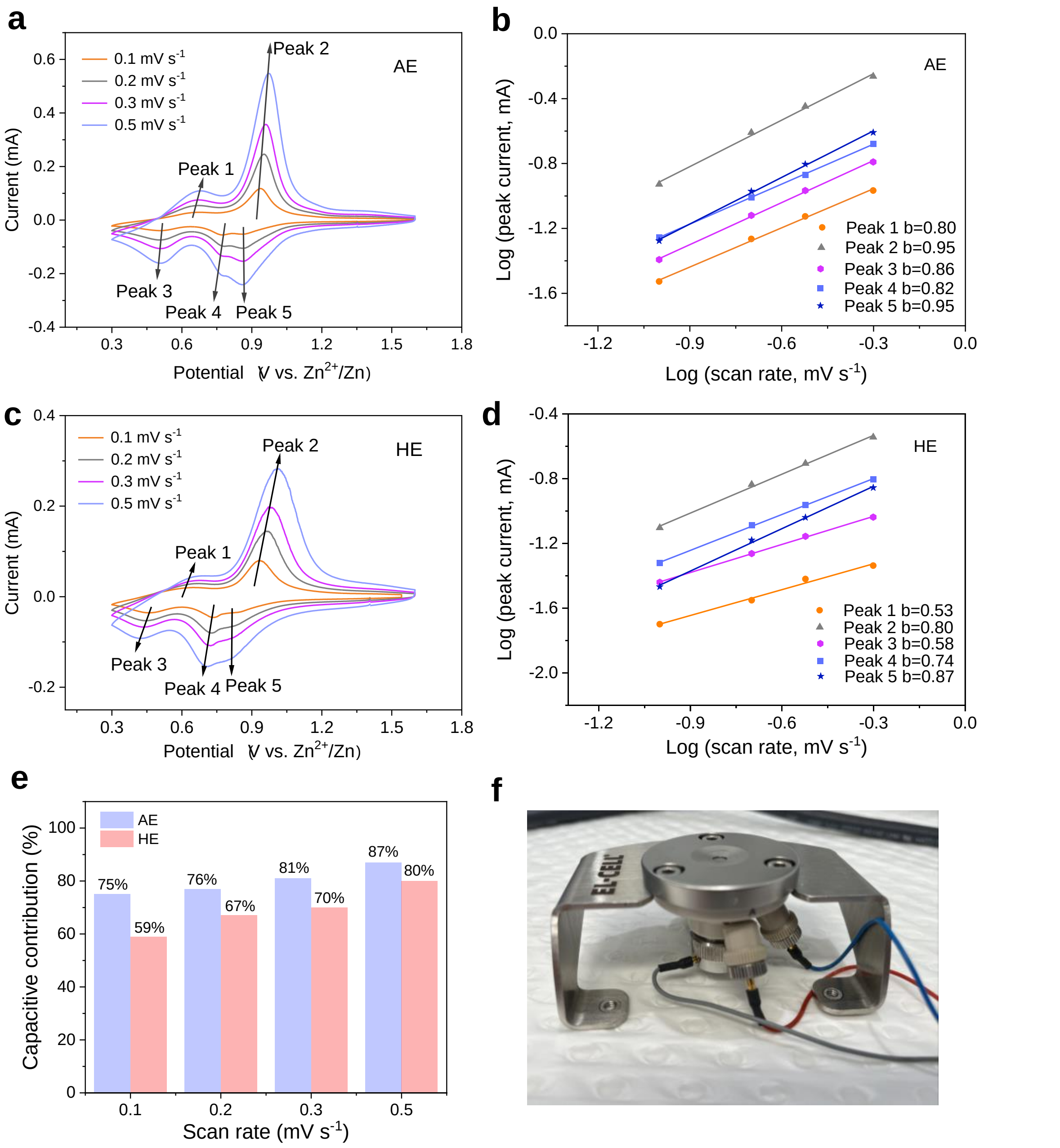

Supplementary Fig. S22. CV characterizations. (a-d) CV curves of NVO electrode at various scan rates and corresponding plots of log (peak current) vs. log (scan rate) in AE and HE, respectively; (e) The comparison of the capacitive contribution at various scan rates in AE and HE, respectively; (f) 3-electrode battery device.

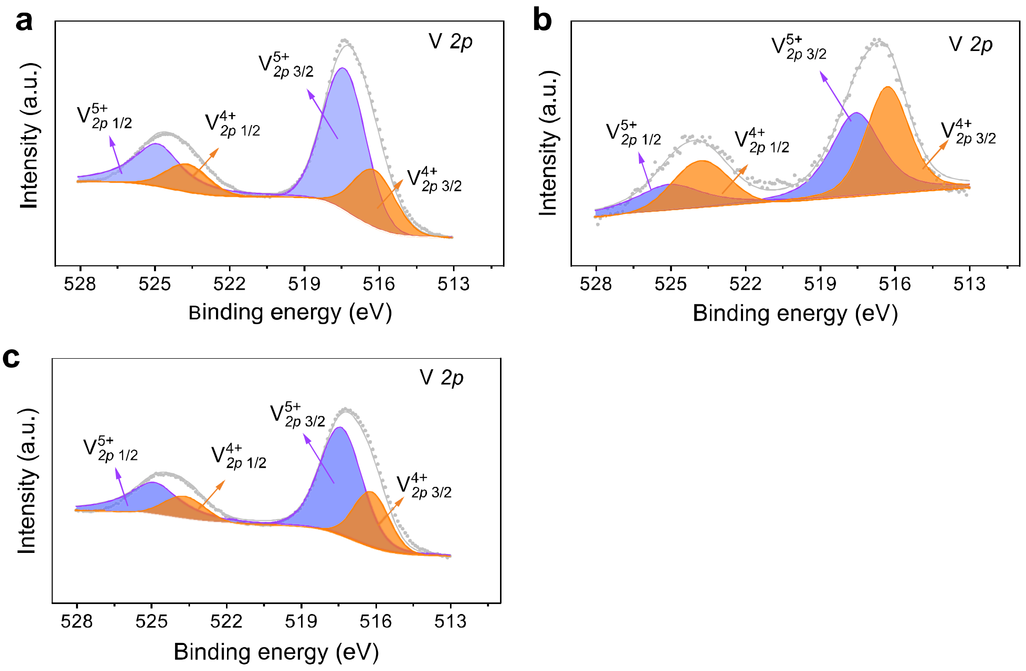

Supplementary Fig. S23. V *2p* XPS spectra for NVO electrodes in (a) pristine, (b) discharged at 0.3 V, and (c) charged back to 1.6 V, state.

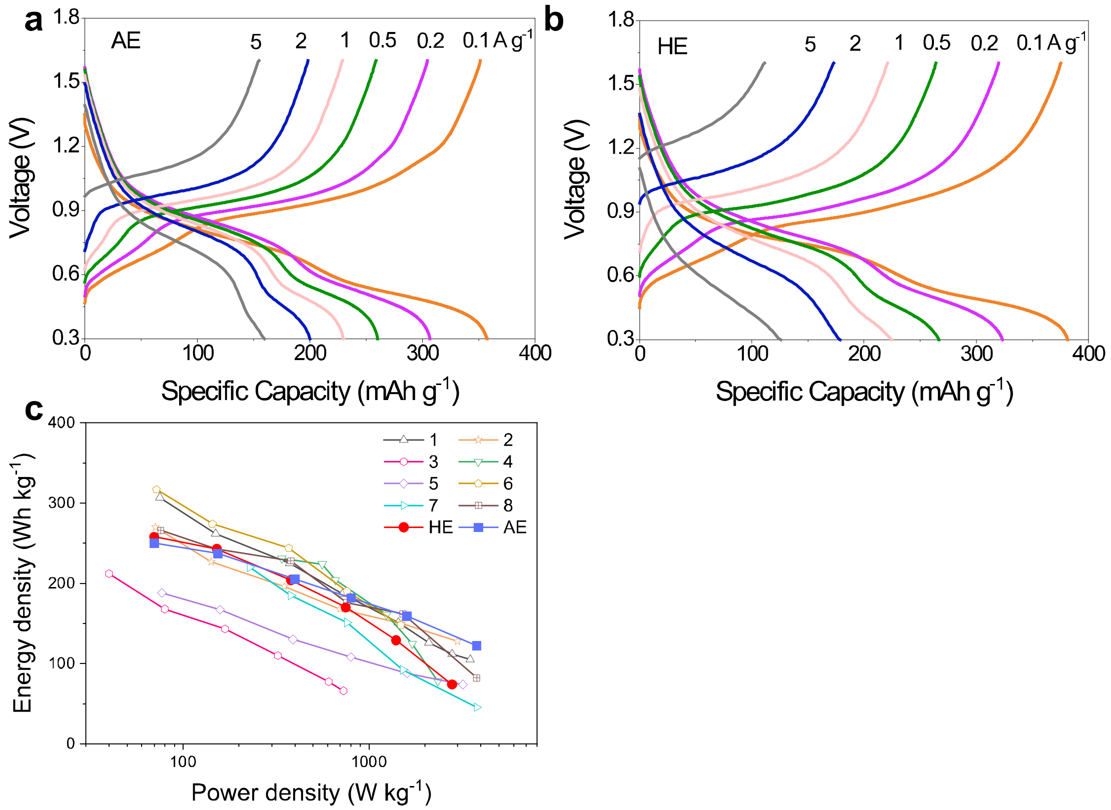

Supplementary Fig. S24. Charge-discharge curves for Zn||NVO battery in (a) AE and (b) HE tested at specific current, respectively, 0.1, 0.2, 0.5, 1, 2 and 5 A g^{-1}. The mass loading of NVO is 1.8 mg cm^{-2}. (c) Comparison of energy density/power density of this work with reported findings based on the mass of cathode active materials.

Supplementary Table S1. Comparison of the energy density and power density for ZIBs based on the mass of cathode active materials.

No.	Cathode material	Electrolyte	Mass loading	Energy density (Wh kg^{-1})	Power density (W kg^{-1})	Specific current (A g^{-1})	Lifespan (cycle)	Reference
1	NVO	1 M $ZnSO_4$/1 M Na_2SO_4 aqueous solution	1.2 mg cm^{-2}	307<br>262<br>225<br>187<br>151<br>126<br>112<br>105	75<br>150<br>375<br>720<br>1440<br>2100<br>2800<br>3500	5	2000	*Science China Chemistry*, 2019, 62, 609–615
2	NVO	1 M $ZnSO_4$/1 M Na_2SO_4 aqueous solution	2 mg cm^{-2}	270<br>227<br>196<br>168<br>150<br>128	71<br>142<br>350<br>700<br>1500<br>3000	1<br>4	100<br>1000	*Nat. Commun.*, 2018, 9, 1656
3	NVO	3 M $ZnSO_4$ aqueous solution	2.4 mg cm^{-2}	212<br>168<br>143<br>110<br>77.5<br>66	40<br>80<br>168<br>324<br>608<br>730	0.05<br>0.4	300<br>2000	*ACS Appl. Mater. Interfaces* 2020, 12, 54627−54636
4	$Na_2V_6O_{16} \cdot 3H_2O$	1.0 M $Zn(ClO_4)_2$ in propylene carbonate	N/A	231<br>224<br>204<br>161<br>125<br>78	340<br>560<br>660<br>1240<br>1710<br>2350	0.5<br>2<br>5	100<br>5000<br>5000	*Batteries & Supercaps* 2020, 3, 254-260
5	$Na_2V_6O_{16} \cdot nH_2O$	0.5m $Zn(ClO_4)_2$ with 18m $NaClO_4$ aqueous solution	3~4 mg cm^{-2}	188<br>167<br>130	77<br>158<br>390	4	2000	*Energy Environ. Sci.*,2021, 14, 4463

				108<br>88<br>74	800<br>1600<br>3200			
6	$Na_5V_{12}O_{32}\cdot11.9H_2O$	3 M $Zn(CF_3SO_3)_2$ aqueous solution	1.1~3.4 mg cm^{-2}	317<br>274<br>244<br>190<br>148	72<br>144<br>370<br>760<br>1480	0.1<br>1	45<br>3800	*Mater. Today Energy*, 2021, 21, 100757
7	$Na_5V_{12}O_{32}$	3 M $ZnSO_4$ aqueous solution	~2 mg cm^{-2}	220<br>185<br>151<br>92<br>45.6	228<br>380<br>760<br>1520<br>3800	0.1<br>0.5<br>4	50<br>100<br>2000	*Adv. Energy Mater.*, 2018, 8, 1801819
8	Mn-Doped $Na_5V_{12}O_{32}$	2 M $ZnSO_4$/0.5 M Na_2SO_4 aqueous solution	~1.5 mg cm^{-2}	266<br>243<br>228<br>176<br>162<br>82	76<br>152<br>380<br>760<br>1520<br>3800	0.1<br>1	50<br>250	*Energy Fuels*, 2021, 35, 13483–13490
9	NVO	AE	~1.8 mg cm^{-2}	248<br>235<br>205<br>182<br>159<br>122	70<br>154<br>400<br>800<br>1600<br>3800	0.1<br>0.5<br>4	100<br>400<br>6000	*This work*
10	NVO	HE	~1.8 mg cm^{-2}	260<br>244<br>204<br>170<br>129<br>74	70<br>152<br>380<br>750<br>1400<br>2800	0.1<br>0.5<br>4	700<br>3000<br>6000	*this work*

Supplementary Table S2. Comparison of the energy density of this work with reported aqueous Li-ion and Na-ion batteries.

No	Cathode/anode electrodes	Cathode/anode mass ratio	Average voltage	Energy density based on the mass of cathode and anode (Wh kg^{-1})	Reference
1	$LiTi_2(PO_4)_3$/C‖ $LiMn_2O_4$	1.5:1	1.55 V	68	*Sci Rep*, 2015, 5, 10733
2	TiS_2‖$LiMn_2O_4$	2:1	N/A	78	*Electrochem. Commun.*, 2017, 82, 71-74
3	VO_2‖$LiMn_2O_4$	N/A	1.5 V	55	*Science*, 1994, 264, 1115-1118
4	LiV_3O_8‖$LiNi_{0.81}Co_{0.19}O_2$	N/A	1~1.2 V	54	*Electrochimica Acta*, 2000, 46, 59–65
5	Mo_6S_8‖$LiMn_2O_4$	2:1	1.5 V and 2 V	84	*Science*, 2015, 350, 938-943
6	acetylene black‖$LiMn_2O_4$	1:2	~1.3 V	35	*J. Electrochem. Soc.*, 2006, 153, A450–A454
7	$LiTi_2(PO_4)_3$‖$LiFeO_4$	1:1	0.9 V	50	*Nature Chemistry*, 2010, 2, 760–765
8	$LiTi_2(PO_4)_3$‖$LiMn_2O_4$	1:1	1.5 V	60	*Adv. Funct. Mater.*, 2007, 17, 3877–3884
9	Polyimide‖$LiCoO_2$	1.2:1	1.12 V	80	*J. Power Sources*, 2014, 249,367–372
10	$Na_3MnTi(PO_4)_3$‖Na3MnTi$(PO_4)_3$	1:1	1.4 V	40	*Angew. Chem. Int. Ed.*, 2016, 55, 12768–12772
11	polymerized pyrene-4,5,9,10-tetraone ‖$Na_3V_2(PO_4)_3$	N/A	0.65 V	30	*Nat. Mater.*, 2017, 16, 841–848
12	Polyimide‖NaI	N/A	0.8 V	63.8	*Sci. Adv.*, 2016, 2, e1501038
13	$NaTi_2(PO_4)_3$‖$Na_{0.44}MnO_2$	2.5:1	1.1 V	33	*Adv. Energy Mater.*, 2013, 3,290–294
14	Zn‖NVO	N/A	0.75	70	*Nat Commun.*, 2018, 9, 1656
15	Zn‖NVO	1:1.8	0.75 V	77	*This work*

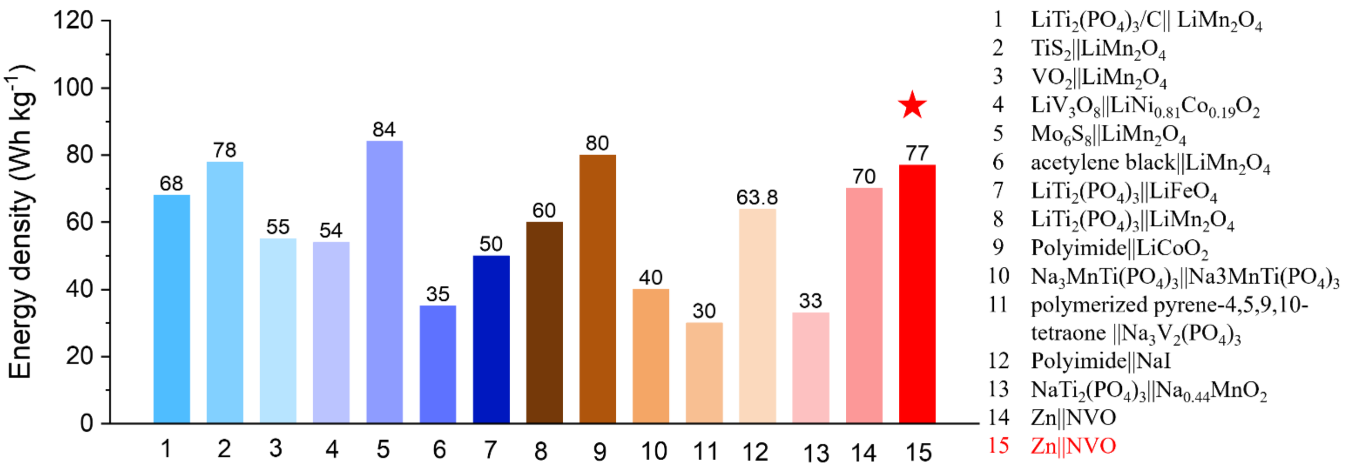

Supplementary Fig. S25. Comparison of energy density for Zn||NVO battery in HE with selected aqueous batteries based on mass of cathode and anode active materials. Detailed data are in **Table S2**.

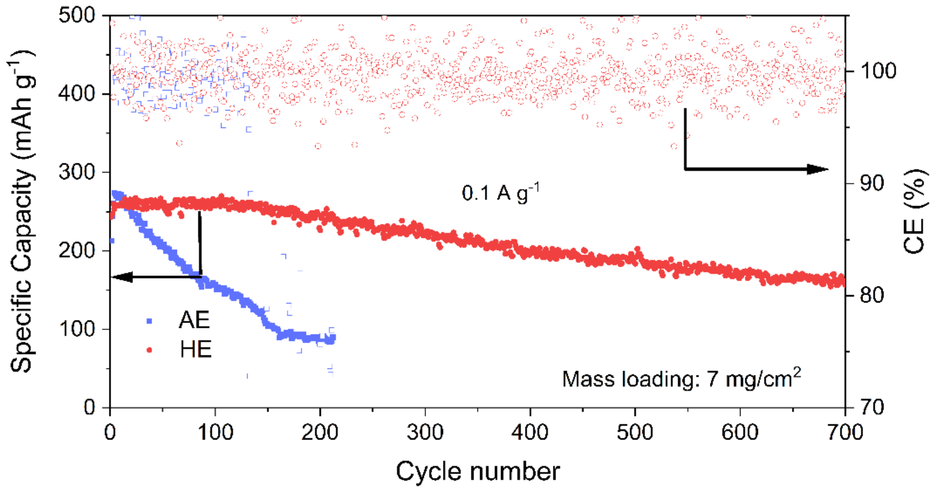

Supplementary Fig. S26. Cyclic stability and CE of Zn||NVO cells tested in AE and HE, respectively, at a specific current of 0.1 A g^{-1}.

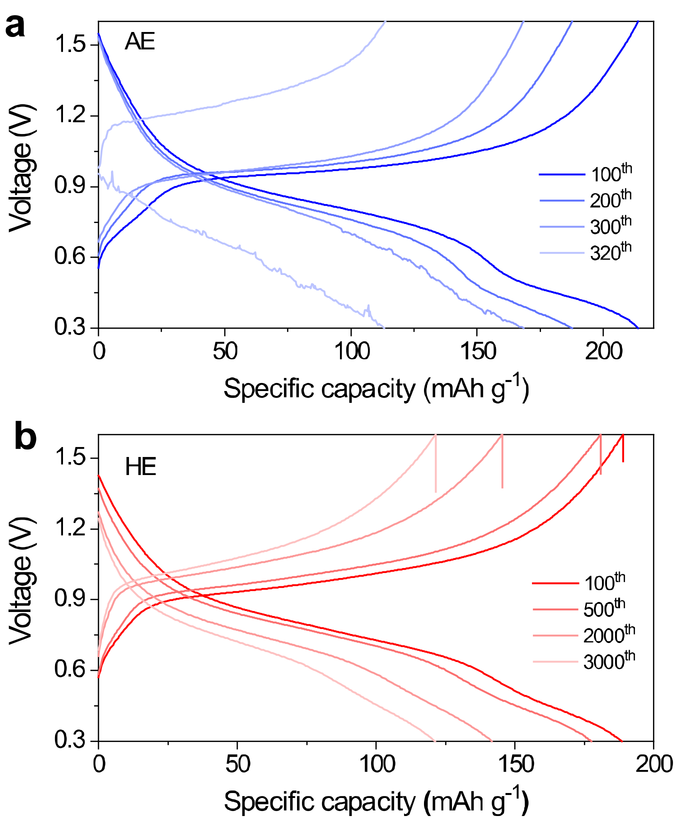

Supplementary Fig. S27. The charge-discharge curves of Zn||NVO battery in (a) AE and (b) HE.

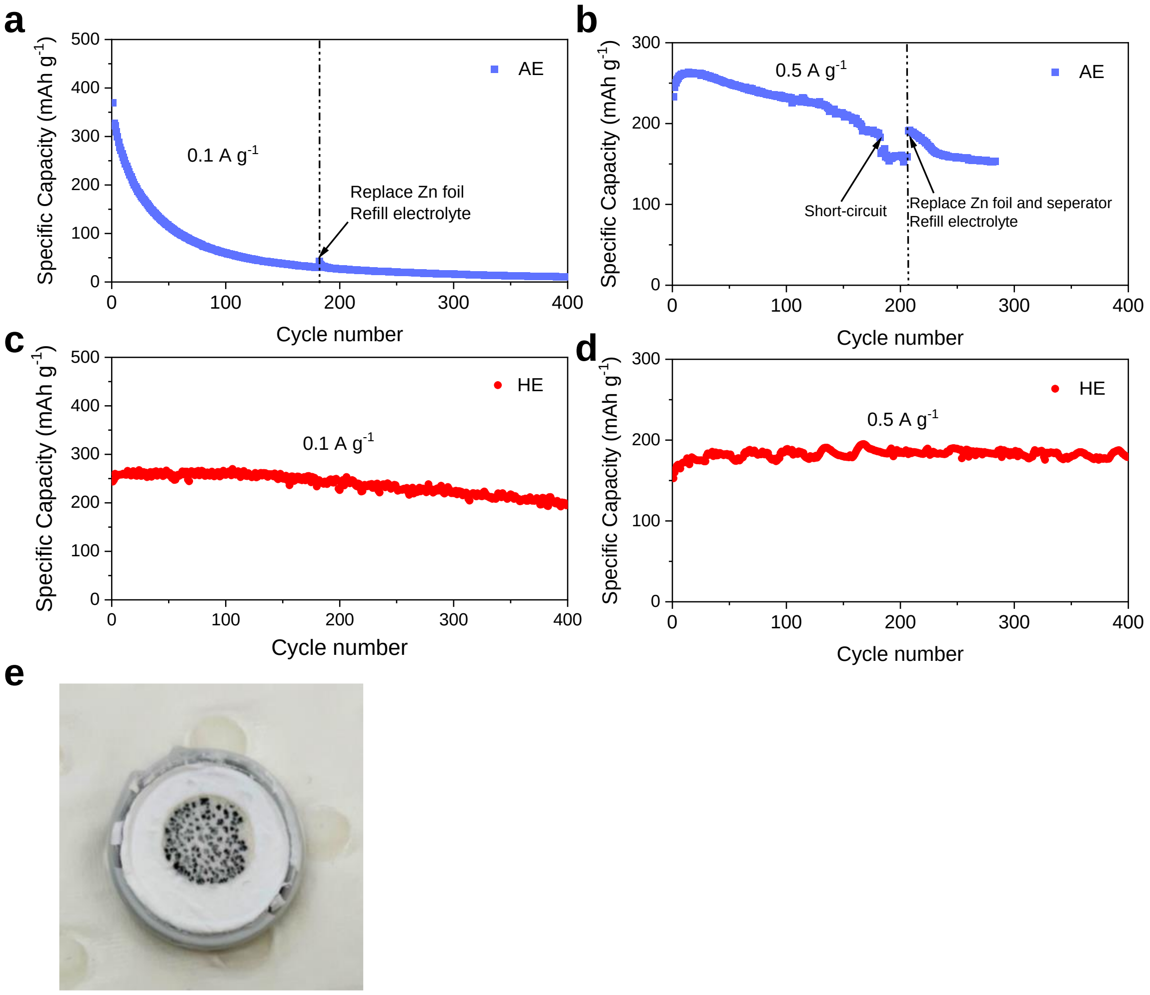

Supplementary Fig. S28. Failure analysis of Zn||NVO batteries tested in AE. (a) The battery was tested at 0.1 A g^{-1}, and the Zn anode was replaced and the electrolyte was refilled at the 182nd cycle; (b) The battery was tested at 0.5 A g^{-1}, and the Zn anode was replaced and the electrolyte was refilled at the 207th cycle; (c) The battery disassembled after short-circuit;(d-e) The reference battery tested in HE.

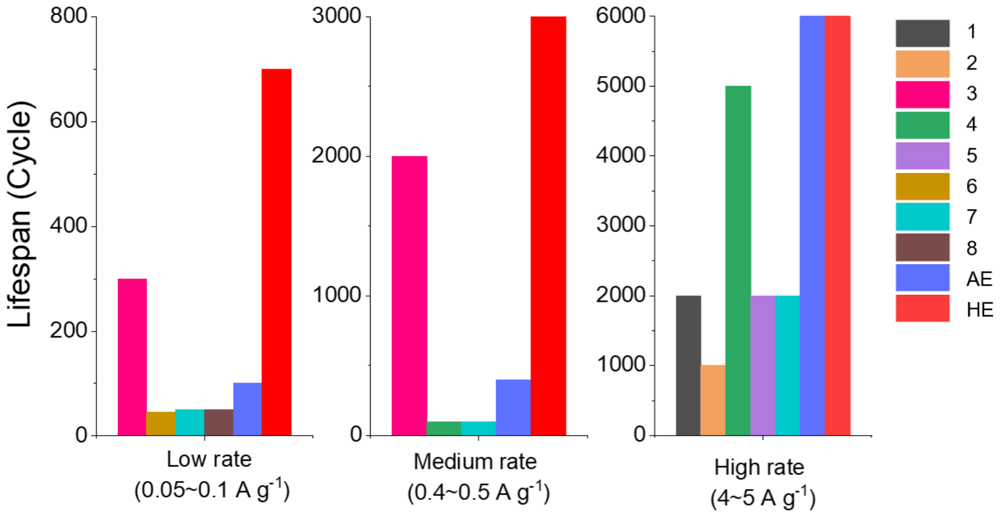

Supplementary Fig. S29. Comparison of battery lifespan with reported results based on data of **Table S1**.

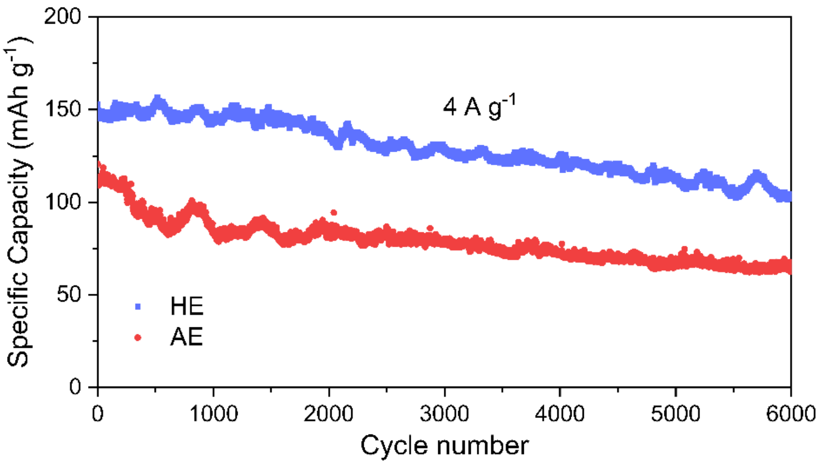

Supplementary Fig. S30. Cyclic stability and CE of Zn||NVO cells tested in AE and HE, respectively, at a specific current of 4 A g^{-1}.

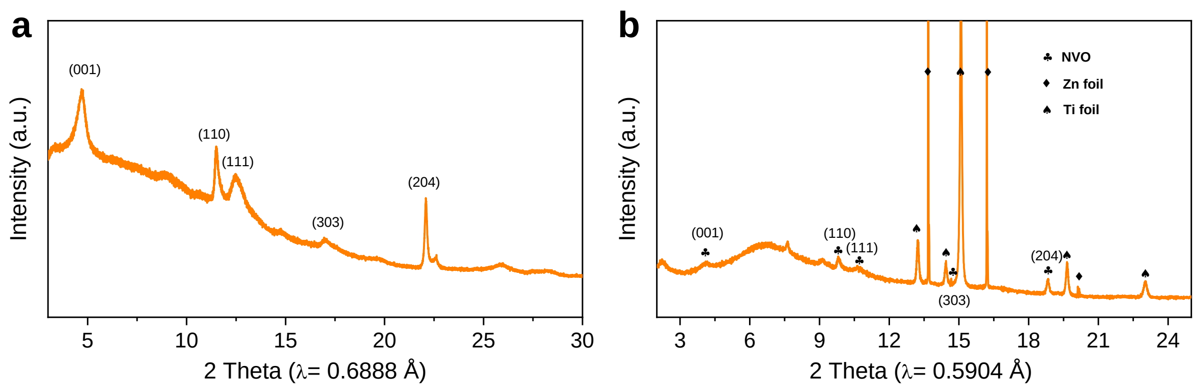

Supplementary Fig. S31. (a) XRPD pattern of the NVO electrode; (b) XRPD pattern of the initial state of the *in operando* Zn||NVO cell.

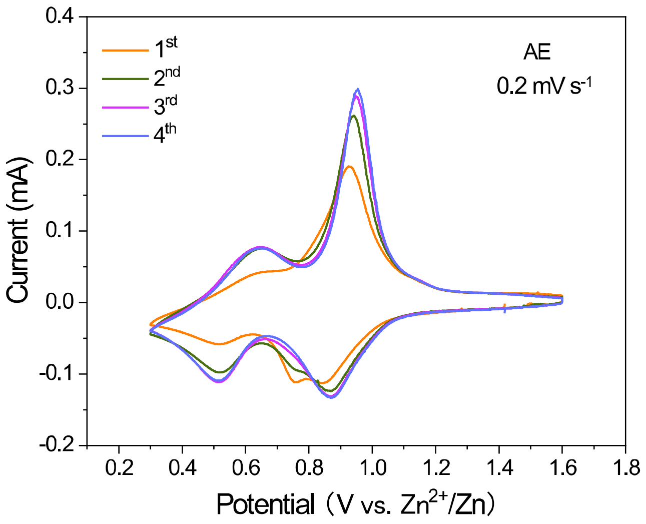

Supplementary Fig. S32. 3-electrode CV curves of NVO electrode tested in AE.

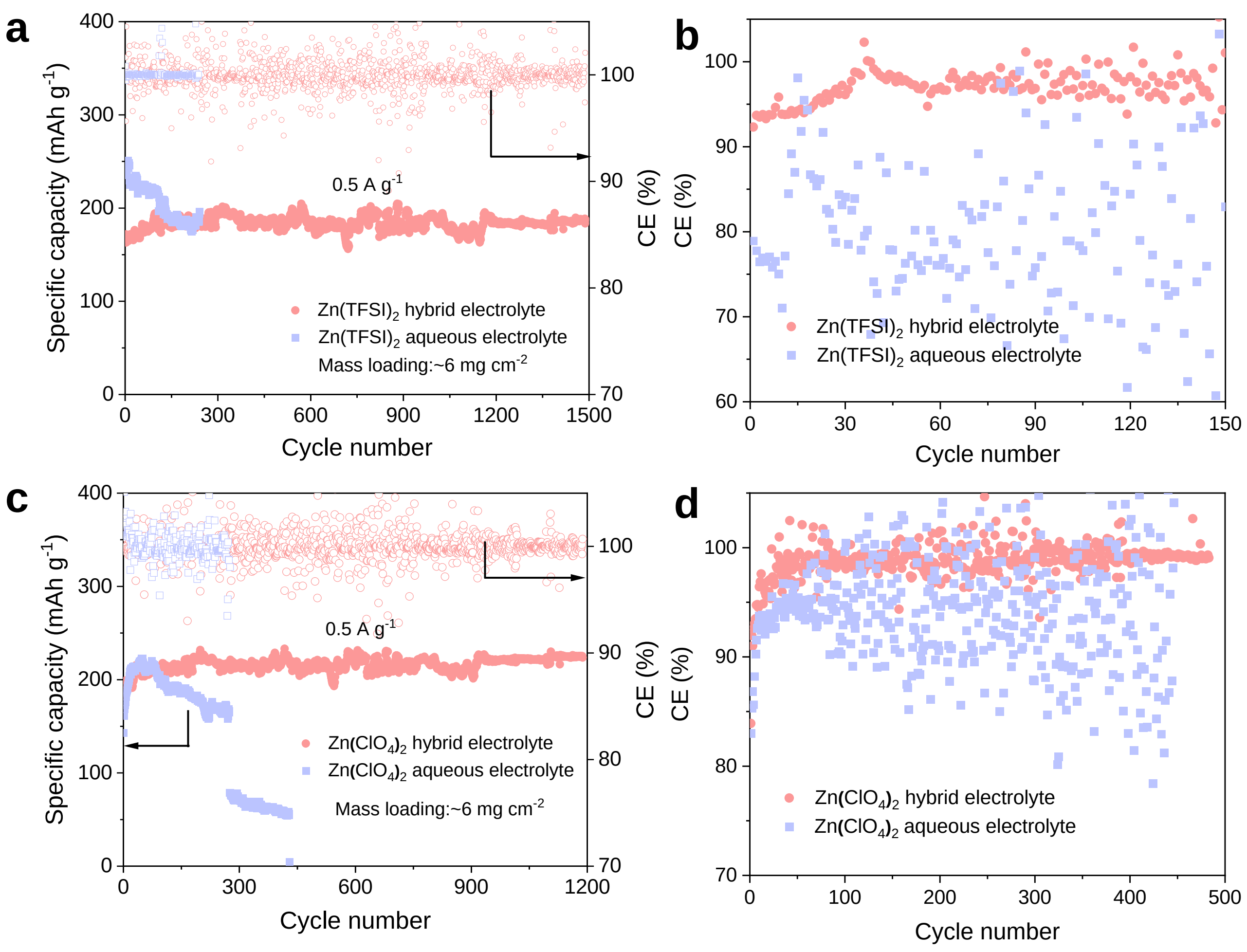

Supplementary Fig. S33. The comparison of the cycling performance of Zn||NVO cells and the CE of Zn platting/stripping tested in hybrid electrolyte and aqueous electrolyte. (a) (b) 1 M $Zn(TFSI)_2$ is used as the salt; (c) (d) 1 M $Zn(ClO_4)_2$ is used as the salt. In the hybrid electrolyte, the solvent is DMAC/TMP/H_2O in a volume ratio of 5:2:3.

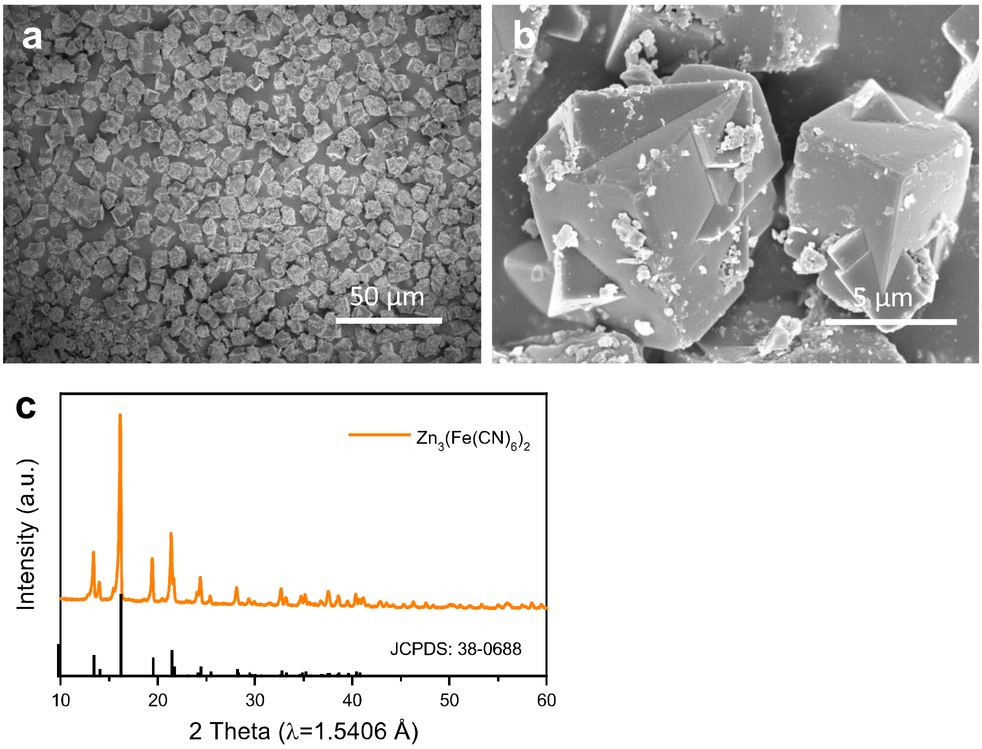

Supplementary Fig. S34. (a-b) The SEM images and (c) The XRD pattern of $Zn_3(Fe(CN)_6)_2$ powders.

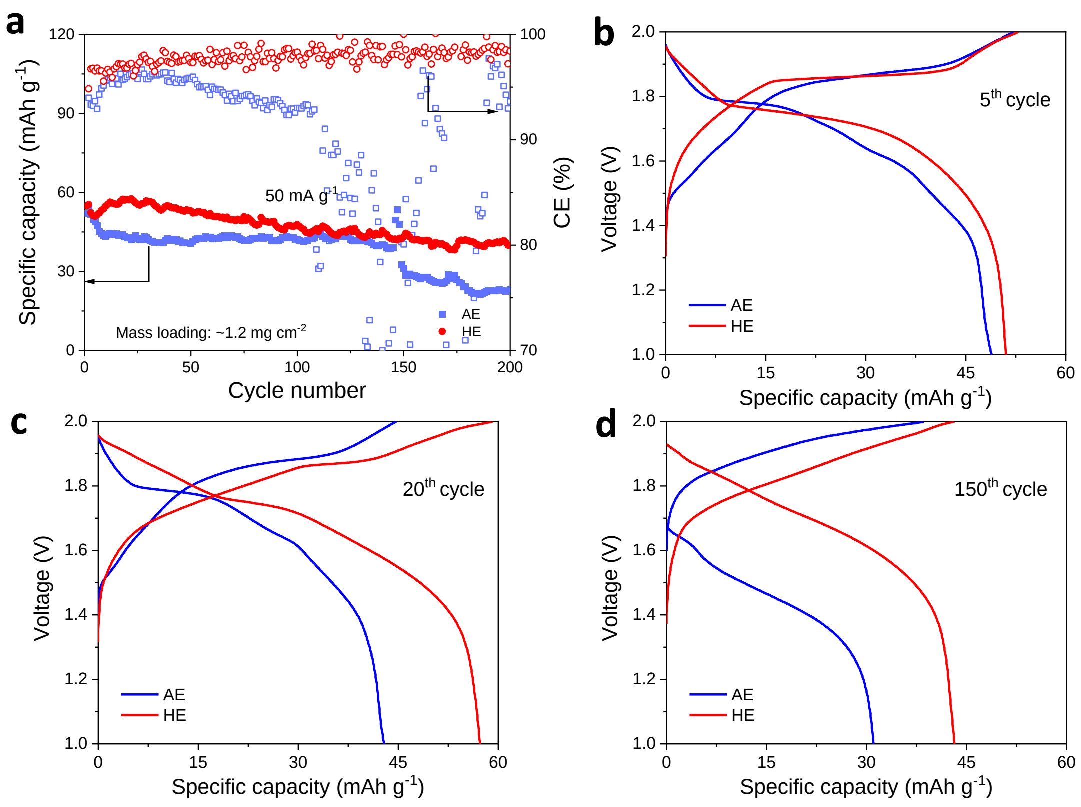

Supplementary Fig. S35. (a) Cycling performance of Zn||$Zn_3(Fe(CN)_6)_2$ cells tested at 50 mA g^{-1} in AE and HE; (b-d) the voltage profiles of the 5th, 20th and 150th cycles, respectively.

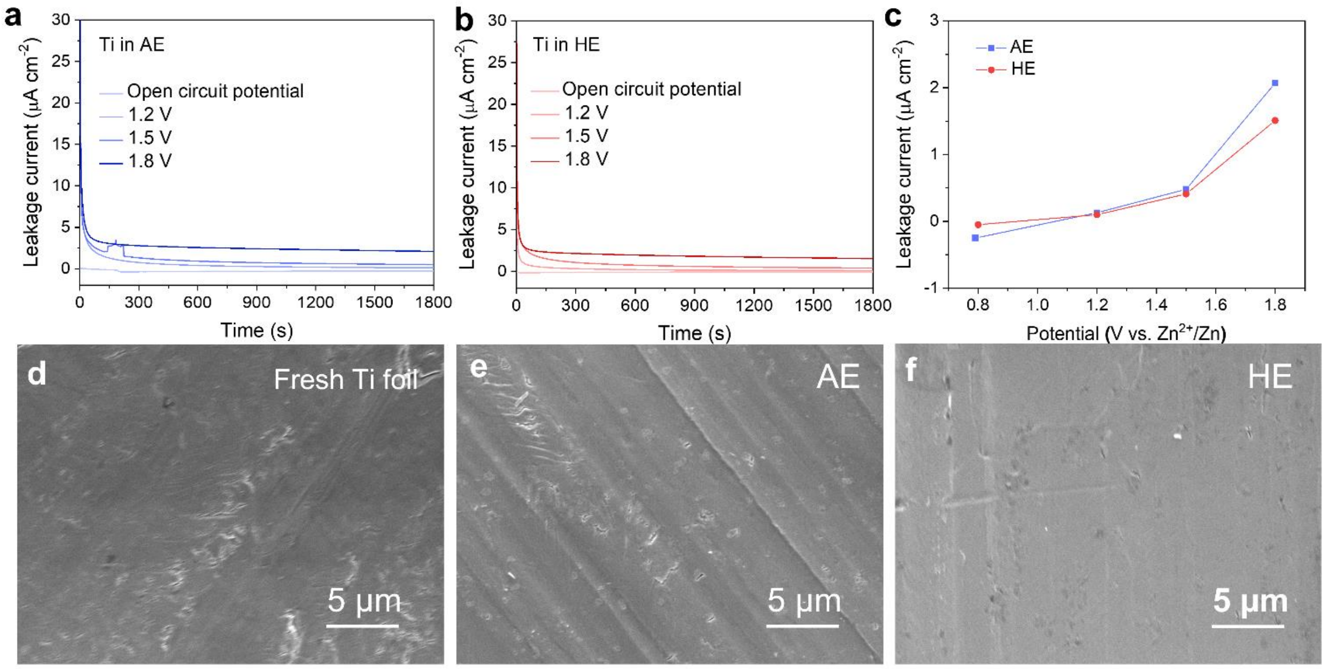

Supplementary Fig. S36. Leakage current for Ti-foil in, (a) AE and (b) HE over time. (c) Stabilized leakage currents for Ti-foil at open-circuit voltage, 1.2, 1.5 and 1.8 V, respectively. SEM images of fresh Ti-foil (d) worked for 300 cycles in (e) AE and (f) HE.

Because during operation of ZIBs, the applied voltage on Ti-foil can be up to 1.8 V, the corrosion of the Ti current collector was therefore determined *via* measuring leakage current during voltage hold. **Fig. S35a-c** evidences that the leakage current remains low for both AE and HE at a high voltage of 1.8 V, with the leakage current for battery in HE less than that in AE, confirming better stability of Ti-foil in HE. SEM images (**Fig. S35d-f**) of the Ti-foil from batteries after 300 cycles exhibit smooth surface(s) with no apparent corrosion.

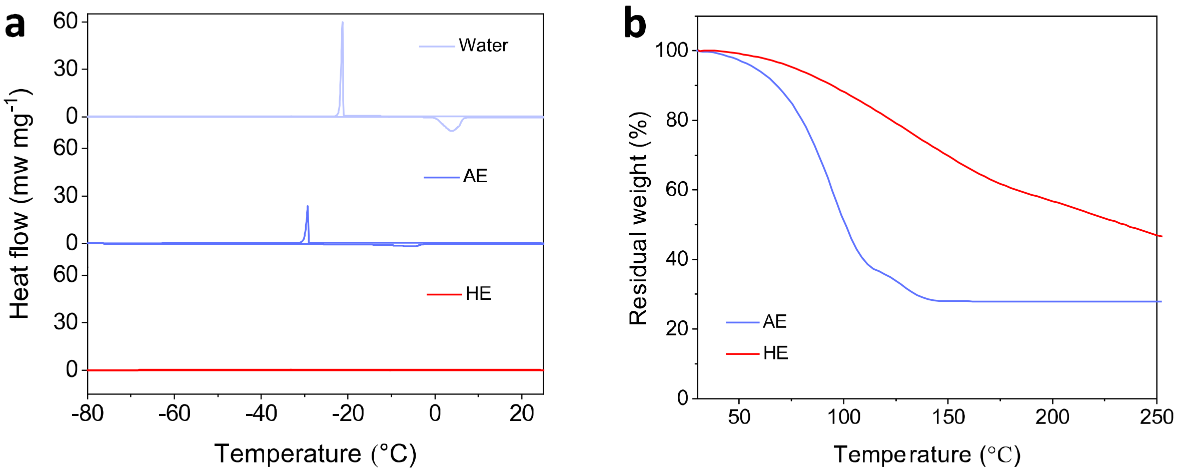

Supplementary Fig. S37. (a) DSC curves of water, AE and HE; (b) TGA curves for AE and HE under N_2 atmosphere using a heating rate of 20 ℃ min^{-1}.

Supplementary Table S3. The ionic conductivity and viscosity of AE and HE.

Temperature	AE		HE	
	Ionic conductivity (mS cm^{-1})	Viscosity mPa s^{-1}	Ionic conductivity (mS cm^{-1})	Viscosity mPa s^{-1}
70 ℃	65.8	0.96	22.1	3.73
50 ℃	58.7	1.16	17.1	5.66
25 ℃	49.1	1.77	9.0	11.65
0 ℃	28.4	3.59	5.1	25.62
−20 ℃	—	—	2.1	155.13
−40 ℃	—	—	0.73	1307

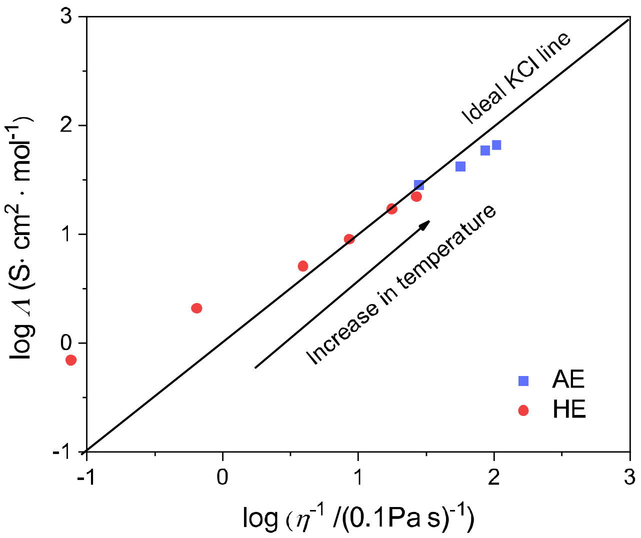

Supplementary Fig. S38. Walden plot of temperature-dependent conductivities and viscosities for AE and HE.

Walden's rule reveals that the product of molar conductivity (Λ) of a liquid solution and its viscosity (η) is a constant at a given temperature, as expressed by the following equation:

$$\Lambda\,\eta = C = \text{Constant} \qquad (1)$$

$$\log \Lambda = \log C + \log \eta^{-1} \qquad (2)$$

The data of a dilute aqueous KCl solution, an ideal solution that all ions are dissociated, can be used as the "ideal" Walden line. In Fig. S38., both the Walden plot of AE and HE are very close to the "ideal" line, indicating that the $Zn(OTf)_2$ salt is well dissociated in these two electrolytes.

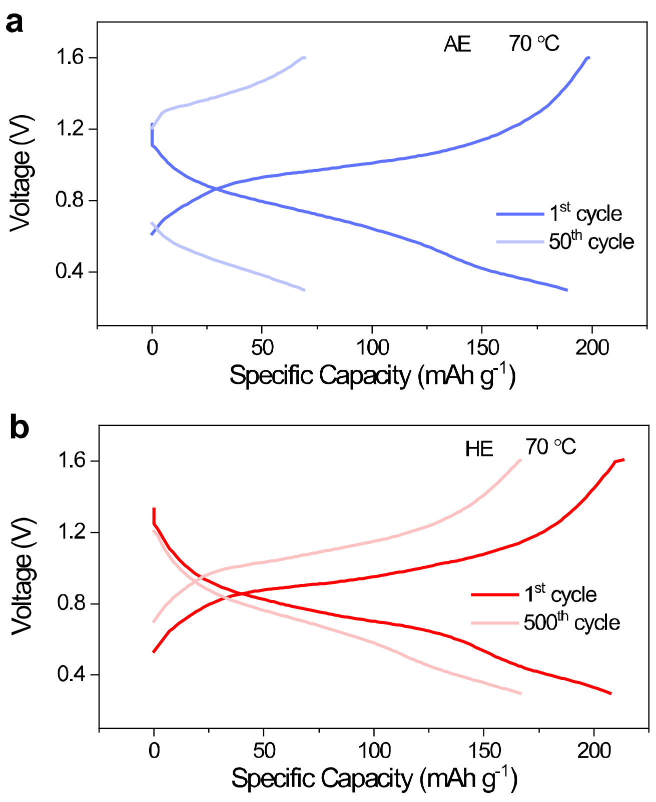

Supplementary Fig. S39. Charge/discharge curve for Zn||NVO battery tested at 70 °C in AE (a) and in HE (b).

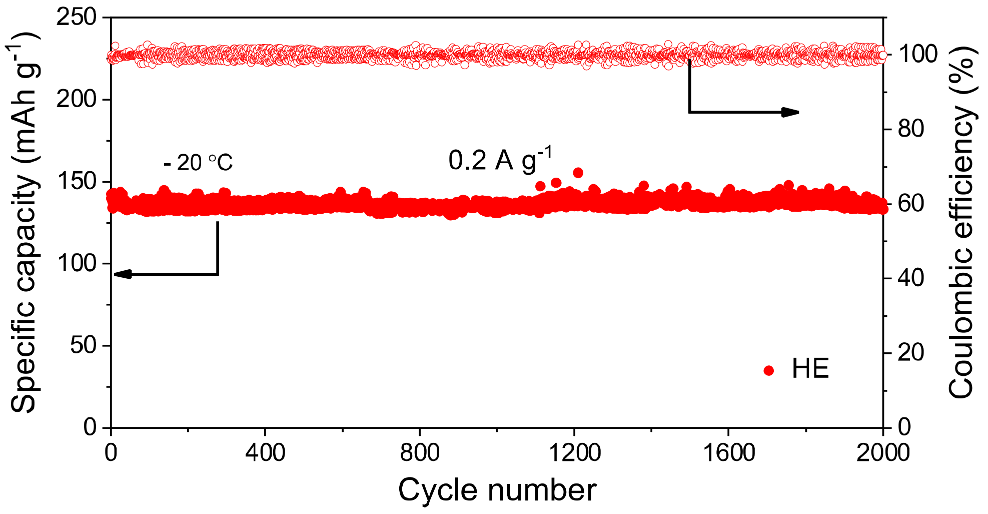

Supplementary Fig. S40. Cyclic performance for Zn||NVO cell at − 20 °C using HE as electrolyte.

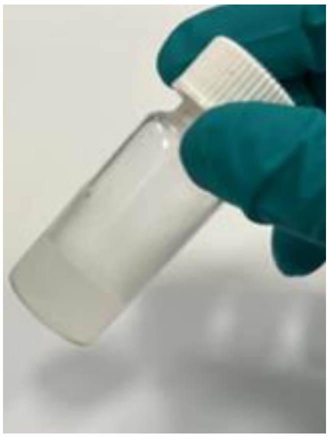

Supplementary Fig. S41. Digital image of frozen AE at a temperature − 20 °C.

Supplementary Table S4. Comparative summary of state-of-art selected electrolytes for ZIBs.

No.	Electrolyte recipe	Flammability (Y/N)	Prefered Zn plating orientation	CE of Zn plating-stripping	Oxidation voltage (vs. Zn^{2+}/Zn)	Cathode	Cycle life	References
1	2 M $Zn(OTf)_2$ + 7 M DEC aqueous electrolyte	N	N	Zn‖Cu cell, 99.24%, 400 cycles, 1 mA cm^{-2}, 1 mAh cm^{-2}	N/A	$V_2O_5 \cdot nH_2O$ (mass loading N/A)	202 mAh g^{-1} (2 A g^{-1}), 5000 cycles (RT)	*ACS Nano*, 2022, 16, 9667−9678.
2	0.5 M $Zn(OTf)_2$ in TMP/DMC	N	N	Zn‖SS cell, 99.15%, 300 cycles, current and capacity N/A	2.25 V	VS_2, 4 mg cm^{-2}	~113 mAh g^{-1} (0.1 A g^{-1}), 500 cycles (RT)	*Adv. Mater*,. 2019, 31, 1900668.
3	3 M $Zn(OTf)_2$ in PC/H_2O (2:8)	N	N	N/A	N/A	$NaV_3O_8 \cdot 1.5 H_2O$, 8 mg cm^{-2}	150 mAh g^{-1} (0.2 A g^{-1}) 400 cycles, −40 °C<br>229 mAh g^{-1} (5 A g^{-1}) 1000 cycles, 30°C	*Adv. Funct. Mater.*, 2022, 32, 2111714.
4	0.5 M $Zn(OTf)_2$ in TEP/H_2O (7:3)	N	N	Zn‖SS cell, 93.71%, 100 cycles, current and capacity N/A	2.25 V	KCuHCf, 1 mg cm^{-2}	~80 mAh g^{-1}, 1000 cycles, average CE 97.66% (RT)	*Angew. Chem. Int. Ed.*, 2019, 58, 2760–2764.
5.	4 M $Zn(TFSI)_2$ + 4 M $P_{444(2O1)}$-TFSI in H_2O	N/A	N	Zn‖Cu cell, ~99%, 1.17 mA cm^{-2}, 1.17 mAh cm^{-2}, 20% Zn utilization, 16 cycles	2.42 V	$Na_2V_6O_{16} \cdot 1.63 H_2O$, 1 mg cm^{-2}	~118 mAh g^{-1}, 1900 cycles (0.3 A g^{-1}) (RT)	*Angew. Chem. Int. Ed.*, 2021, 60, 12438–12445.
6	1 M $Zn(OTf)_2$ in PC/H_2O (5:5)	N	Y	Zn‖Cu cell, 99.93%, 1 mA cm^{-2}, 0.5 mAh cm^{-2}, 500 cycles	~ 2.5 V	PANI, 1.5-2 mg cm^{-2}	~75 mAh g^{-1} (0.2 A g^{-1}) 200 cycles, −20 °C<br>~100 mAh g^{-1} (0.2 A g^{-1}) 200 cycles, 50 °C	*J. Am. Chem. Soc.*, 2022, 144, 7160−7170.
7	3 M $ZnSO_4$ + 10 mM α-cyclodextrin aqueous electrolyte	N	Y	Zn‖Cu cell, ~99.9%, 1 mA cm^{-2}, 1 mAh cm^{-2}, 600 cycles	~ 2.5 V	V_2O_5, 6.4 mg cm^{-2}	~250 mAh g^{-1}, 200 cycles (1 A g^{-1}) (RT)	*J. Am. Chem. Soc.*, 2022, 144, 11129−11137.
8	2 M $ZnSO_4$ and 0.0085 M La(NO3)3) aqueous electrolyte	N	Y	Zn‖Ti cell, ~99.9%, 2 mA cm^{-2}, 1 mAh cm^{-2}, 2200 cycles	N/A	VS_2, 8 mg cm^{-2} (N/P ratio 4:3)	120 mAh g^{-1} (0.1 A g^{-1}), 100 cycles (RT)	*Nat. Commun.*, 2022, 13, 3252.
9	1 mol/Kg $Zn(TFSI)_2$ + 20 mol/kg LiTFSI aqueous electrolyte	N	N	Zn‖Pt (three electrode cell), 99.7%	N/A	$LiMn_2O_4$, 2.4 mAh cm^{-2}	38 mAh g^{-1} (4 C), 4000 cycles (RT)	*Nat. Mater.*, 2018, 17, 543–549.

10	2 M $ZnSO_4$ in NMP/H_2O (5:5)	N/A	Y	Zn‖Cu cell, ~99.7%, 1 mA cm^{-2}, 0.5 mAh cm^{-2}, 1000 cycles	N/A	VS_2, 5.3 mg cm^{-2}	125 mAh g^{-1} (1 A g^{-1}), 2000 cycles (RT)	*Adv. Energy Mater.*, 2022, 12, 2103231.
11	3 M $ZnSO_4$ + 0.5 M glycine aqueous electrolyte	N	Y	Zn‖Cu cell, 99.68%, 2 mA cm^{-2}, 2 mAh cm^{-2}, 650 cycles	N/A	$NH_4V_4O_{10}$ (mass loading N/A)	350 mAh g^{-1} (5 A g^{-1}), 3000 cycles (RT)	*ACS Nano, 2022,* DOI: 10.1021/acsnano.2c09317
12	1 M $ZnAc_2$ + 4 M NH_4I aqueous electrolyte	N	N	Zn‖Cu cell, 99.8%, 1 mA cm^{-2}, 1 mAh cm^{-2}, 100 cycles	N/A	I_2 (mass loading N/A)	~1.1 mAh cm^{-2}, 200 cycles (RT)	*J. Am. Chem. Soc.*, 2022, 144, 18435−18443
13	1.3 M $ZnCl_2$ in H_2O/DMSO (volume ratio of H_2O/DMSO = 4.3:1)	N/A	N	Zn‖Cu cell, 99.5%, 1 mA cm^{-2}, 0.5 mAh cm^{-2}, 400 cycles	~2.2 V	MnO_2 (mass loading N/A)	150 mAh g^{-1} (8 C), 500 cycles (RT)	*J. Am. Chem. Soc.*, 2020, 142, 21404−21409.
14	BMITFSI:Zn$(TFSI)_2$ (water/ionic liquid mass 20%)	N	N	Zn‖Cu cell, 99.27%, 1 mA cm^{-2}, 0.5 mAh cm^{-2}, 400 cycles	~2.5 V	PANI, 0.8-0.95 mg cm^{-2}	~60 mAh g^{-1} (1 A g^{-1}), 1000 cycles (RT)	*ACS Energy Lett.*, 2023, 8, 608−618.
15	2 M $ZnSO_4$ in water/EG (volume ratio of EG 40%)	N	N	Zn‖Ti cell, ~98%, 2 mA cm^{-2}, 1 mAh cm^{-2}, 120 cycles	~2.8 V	PANI/V_2O_5, 2.5-3.0 mg cm^{-2}	~45 mAh g^{-1} (0.2 A g^{-1}) 6500 cycles, −20 °C<br>~60 mAh g^{-1} (5 A g^{-1}) 50000 cycles, 20 °C	*Energy Environ. Sci.*, 2020, 13, 3527—3535.
16	1 M ZnTFMS/DMF	Y	N	Zn‖SS cell, ~99.8%, 1 mA cm^{-2}, 1 mAh cm^{-2}, 200 cycles	~2.4 V	PQMCT (mass loading N/A)	~22 mAh g^{-1} (0.2 A g^{-1}), 1 cycle, −70 °C<br>~180 mAh g^{-1} (2 A g^{-1}) 1 cycle, 150 °C	*Angew. Chem. Int. Ed.*, 2020, 59, 14577 – 14583
17	7.5 mol/Kg $ZnCl_2$-based aqueous electrolyte	N/A	N	Zn‖Cu cell, ~99%, 0.2 mA cm^{-2}, 0.2 mAh cm^{-2}, 50 cycles	~1.9 V	PANI, (mass loading N/A)	~75 mAh g^{-1} (0.2 A g^{-1}) 2000 cycles, −70 °C	*Nat. Commun.*, 2022, 11, 4463.
18	4 M Zn$(BF_4)_2$ aqueous electrolyte	N	N	Zn‖SS cell, ~95%, 0.5 mA cm^{-2}, 0.5 mAh, 1 cycle	~2 V	TCBQ2, 1-2 mg	~105 mAh g^{-1} (1 C) 1000 cycles, −30 °C<br>~90 mAh g^{-1} (0.1 C) 50 cycles, −30 °C	*J. Mater. Chem. A*, 2021, 9, 7042–7047.
19	2 M Zn$(OTf)_2$ in water/DMC (volume ratio 4:1)	N	N	Zn‖Ti cell, ~99.8%, 1 mA cm^{-2}, 1 mAh cm^{-2}, 200 cycles	N/A	V_2O_5, 2 mg cm^{-2}	~405 mAh g^{-1} (2 A g^{-1}) 1000 cycles (RT)	*Chem. Sci.*, 2021, 12, 5843–5852.
**20**	2.5 M Zn$(NO_3)_2$ +13 M $LiNO_3$in DMAC/H_2O	N/A	N	N/A	N/A	$LiMn_2O_4$, 3 mg cm^{-2}	95 mAh g^{-1} (1 C), 200 cycles (RT)	*ACS Appl. Mater. Interfaces*, 2021,

								13, 39, 46634–46643.
21	*1 M $Zn(OTf)_2$ in DMAC/TMP/H_2O (volume ratio 5:2:3)*	*N*	*Y*	*Zn||Cu cell, 99.5%, 1 mA cm^{-2}, 1 mAh cm^{-2}, 2000 cycles*	*~2.25 V*	*NVO ~6 mg cm^{-2} for RT testing; ~0.6 mg cm^{-2} for high-low temperature testing*	*~264 mAh g^{-1} (0.1 A g^{-1}) 700 cycles, (RT); ~190 mAh g^{-1} (0.5 A g^{-1}) 3000 cycles, (RT); ~100 mAh g^{-1} (0.1 A g^{-1}) 1800 cycles, −40 °C; ~200 mAh g^{-1} (5 A g^{-1}) 1400 cycles (retention ~60%), 70 °C;*	*This work*
